# Cord Leptin, C-Peptide and Insulin Levels in Large for Gestational Age Newborns in Sri Lanka

**DOI:** 10.1155/2019/4268658

**Published:** 2019-12-16

**Authors:** Mawanane Hewa Aruna Devapriya De Silva, Ruwani Punyakanthi Hewawasam, Mampitiya Arachchige Gayani Iresha

**Affiliations:** ^1^Department of Paediatrics, Faculty of Medicine, University of Ruhuna, Sri Lanka; ^2^Department of Biochemistry, Faculty of Medicine, University of Ruhuna, Sri Lanka; ^3^Department of Obstetrics and Gynaecology, Faculty of Medicine, University of Ruhuna, Sri Lanka

## Abstract

**Background:**

Large for gestational age (LGA) infants are more prone to be obese and are at a higher risk of metabolic complications later in life. It is established that Asians have lower skeletal muscle mass and excess body fat for a given body mass index. Thus, objective of this study was to determine the relationship between leptin, insulin, C-peptide in cord blood on the birth weight of newborns and to determine whether these parameters are deviated from data already published from other populations.

**Methods:**

Umbilical cord blood was collected from 90 newborns (male 50, gestational age 38–42 weeks) which comprise of 43 LGA and 47 appropriate for gestational age (AGA) newborns. Serum leptin, insulin and C-peptide levels were measured and anthropometric parameters of the newborn and maternal characteristics were recorded.

**Results:**

Significantly higher (*P* < 0.001) concentrations of leptin, insulin and C-peptide levels (12.670 ± 2.345 ng/mL, 18.725 ± 0.644 *µ*IU/mL, 9.318 ± 0.772 ng/mL) were observed in the LGA group compared to AGA group (7.108 ± 0.906 ng/mL, 13.081 ± 0.428 *µ*IU/mL, 5.439 ± 0.192 ng/mL) and all three parameters showed positive and significant correlations with anthropometric parameters of the newborn and maternal characteristics.

**Conclusion:**

Although increased leptin, insulin and C-peptide levels may be involved in insulin resistance, increased adiposity and macrosomia, they were not significantly deviated from published data from other populations. Other factors may contribute to higher fat mass found in Asian populations and finding this relationship during neonatal period is useful to predict risk factors for childhood obesity.

## 1. Introduction

Childhood obesity has increased to pan-epidemic proportions in the recent past along with a collateral increase in obesity associated morbidity. The etiology of childhood obesity is complex and multifactorial [1]. There is evidence to suggest that the development of obesity and its co-morbidities may be influenced by not only genetic, metabolic, nutritional, socioeconomic and psychological factors but also intrauterine factors [2]. Processes which occur during the prenatal development such as rapid cellular growth, replication, and maturation of organs are sensitive to disturbances in the intrauterine milieu. Also, scientific evidence has emerged to support a hypothesis related to the developmental origins of health and disease. This hypothesis discusses how the intrauterine environment is important in the prenatal programming towards childhood obesity and metabolic dysregulation later in life. According to Hermann et al., experimental evidence to support this hypothesis has also emerged which suggests that the infants who are born large-for-gestational-age (LGA) are more prone to be obese in childhood and adolescence and they are at a higher risk of cardiovascular and metabolic complications related to obesity later in life [3].

During the third trimester of gestation, body composition and the weight of a fetus changes drastically. Thus, over 90% of fetal body fat is deposited and more than fourfold increase in fetal weight is observed during this period [4]. Total body fat at birth is positively associated with birthweight and it reflects the intrauterine growth. According to Patenaude et al., increased neonatal adiposity is associated with childhood obesity [5] and it is further associated with risk factors such as high blood pressure, hyperlipidaemia and hyperinsulinaemia [6]. However, mechanisms which control intrauterine growth are still poorly understood, but adipose tissue was recently recognized to play a major role in linking the fetal growth to the subsequent development of metabolic syndrome during adulthood.

Over the years, a lot of evidence has emerged on pre- and perinatal predictors of childhood adiposity. Some of the examples, as reported by Parker et al. include maternal smoking, excessive gestational weight gain, pre-pregnancy body mass index, gestational glucose tolerance, low adiponectin and high leptin levels in umbilical cord blood at delivery [7]. It was also reported previously that in infants born LGA, leptin levels in cord blood are strongly correlated to birth-weight and insulin levels, suggesting that part of insulin's effect on birth-weight is mediated by leptin [8].

Adipose tissue produces and secretes a number of hormones collectively called as adipocytokines which are important in modulating metabolism and energy homeostasis. Leptin which was identified as a product of obesity (ob) gene is an important member of this adipocytokine family. According to Tung et al., cord blood leptin level has been positively correlated with fetal adiposity at birth [9]. Experimental evidence also emerged to suggest that leptin is also produced by the feto-placental unit, which suggests that trophoblasts and the amnion cells are the nonadipose tissue sources [10]. Therefore, in addition to its function as an indicator of fetal adipose tissue mass, the evidence that support the early appearance of this hormone during fetal development and the recognition of the placenta as another source of leptin production suggest that leptin is important in fetal growth [11].

Mitanchez established the link between maternal glucose homeostasis and the fetal pancreatic response [12]. It has already been reported that glucose crosses the placenta but insulin cannot. Therefore, maternal hyperglycaemia give rise to a subsequent fetal hyperglycaemia resulting in increased birthweight [13].

In recent years, there is accumulating evidence to suggest that the relationship between BMI and percentage body fat differs between ethnic groups. According to Wang et al., Asians' have a lower BMI but a higher percentage body fat compared to Caucasians of the same age group [14]. However, out of 242 participants recruited to the study conducted by Wang et al. 225 were Chinese and rest of the participants were from Japan, Philippines and Korea. None of the participants were from South Asia. In order to understand the relationship between metabolic determinants of obesity in the intrauterine environment and the birth of large for gestational age infants, it is important to understand whether these parameters vary among different ethnic groups including those in the Indian subcontinent. Since percentage body fat is scientifically proven to be different among ethnic groups, it can be predicted that the levels of adipocytokines such as leptin and biomarkers of insulin resistance such as insulin and C-peptide may also be different among ethnic groups and the mechanisms in determining the body weight of a newborn may also be different. Therefore, the objective of the present study was to determine the relationship between adipocytokines such as leptin and metabolic determinants of insulin resistance such as insulin and C-peptide in cord blood on the birth weight of newborns born in a tertiary care centre in Southern Sri Lanka and to determine whether these parameters are deviated from the data already published from other populations.

## 2. Materials and Methods

### 2.1. Subjects

A hospital based cross sectional study was conducted between May 2017 and November 2017 at the professorial unit, Teaching Hospital, Mahamodara, Sri Lanka. The study protocol was reviewed and approved by the Ethical Review Committee of the Faculty of Medicine, University of Ruhuna, Sri Lanka. The objective of the study was explained to each pregnant woman and written informed consent was obtained before enrollment to the study. Term (37 weeks ≤ gestational age ≥ 41 weeks) neonates born appropriate for gestational age (birth weight between 10^th^ and 90^th^ percentile for age and gender based on the CDC 2000 growth charts) and large-for-gestational-age (birth weight above the 90^th^ percentile for age and gender based on the CDC 2000 growth charts) who did not suffer from any complication were recruited to the study. Mothers who had singleton pregnancies and uncomplicated deliveries were considered to obtain the consent. Infants who were small for gestational age (weight for gestation less than 10^th^ percentile) were regarded as high-risk newborns and were not included in this study. Additionally, infants with anomalies or who required intensive care were also excluded. Children who were exposed to maternal chronic disease (including diabetes mellitus or gestational diabetes mellitus) and preeclampsia and children with neonatal respiratory distress syndrome i.e children with an Apgar score of <7 at 5 min were also excluded.

### 2.2. Data Collection

90 mother-child pairs were included in the study. A data extraction sheet was used to extract maternal, obstetric and perinatal data from hospital records. Maternal characteristics such as weight and maternal BMI before pregnancy, weight gain during pregnancy, number of children were recorded. Anthropometric indices such as infant weight and length were measured within 24 hours after delivery. Each measurement was taken by a trained personnel in triplicate and the average result was recorded. Birth weight was determined using a calibrated electronic scale with an accuracy of 2 g. The length was measured with the infant in the supine position with an infantometer containing a stationary headboard, a movable footboard and a built in centimeter scale. Occipital frontal head circumference, chest, mid upper arm, abdominal, hip, thigh and calf circumferences were measured using a nonstretchable tape. The gestational age was estimated by the last menstrual period, confirmed by ultrasonography before 20 weeks of gestation. Symmetry was evaluated by Rohrer's Ponderal Index (PI = birth weight (g)/birth length (cm)^3^ × 100), where infants with PI 2.25 < PI < 3.1 were considered symmetric.

Newborns were divided into two groups according to the birth size. Specifically, birth weights above the 90^th^ percentile or 2 standard deviation score (SDS) above the mean for gestational age were classified as large-for-gestational-age (LGA) and birth weights between the 10^th^ and 90^th^ percentiles were classified as appropriate for gestational age (AGA).

### 2.3. Estimation of Biochemical Parameters

5 ml of cord blood samples were collected after double clamping the umbilical cord before separation of the placenta into ethylenediamine tetraacetic acid (EDTA) tubes immediately after delivery and were stored at 4°C up to a maximum of 12 h. They were then centrifuged, and the plasma was aliquoted and stored at −70°C until assayed.

Leptin and insulin concentrations were measured by enzyme linked immunosorbent assay (ELISA). Fetal insulin resistance was estimated via cord blood C-peptide estimation using ELISA technique.

### 2.4. Statistical Analysis

Statistical analysis was performed using Statistical Package for the Social Sciences (SPSS) 16.0 for Windows software. Data were expressed as mean ± standard deviations (SD), number (percentages) or frequency. Differences in categorical variables were tested using a chi-squared test. The relationship between leptin, insulin, C-peptide and anthropometric indices were assessed by Pearson's correlation while Kruskal–Wallis test was used to assess the significance between parameters. The multiple logistic regression analysis examined influences of maternal parameters on biochemical parameters in cord blood as covariates on the birth of large for gestational age infants. Statistical difference was defined as *p* < 0.05.

## 3. Results

The characteristics of the mother and the newborn enrolled in the study are shown in Table 1. Study population consisted of 90 neonates (50 males and 40 females, gestational age 38–42). Birth weight (*P* ≤ 0.001), birth length (*P* = 0.001) and ponderal index (*P* = 0.005) were significantly higher in the LGA infants compared to the AGA infants. However, a significant difference was not observed in the gestational age at delivery between the two groups but all the circumferences except the calf and abdominal circumferences measured between the two groups were statistically significant. When maternal characteristics were considered, weight (*P* = 0.002) and BMI before pregnancy (*P* = 0.012) were significantly different between LGA and AGA groups.  Table 2 shows characteristics of study subjects according to the sex. A significant difference between sexes was observed only in birth weight and chest circumferences. Although notable differences were observed in the leptin and C-peptide concentrations between male and female newborns, they were not statistically significant.

**Figure 1:**
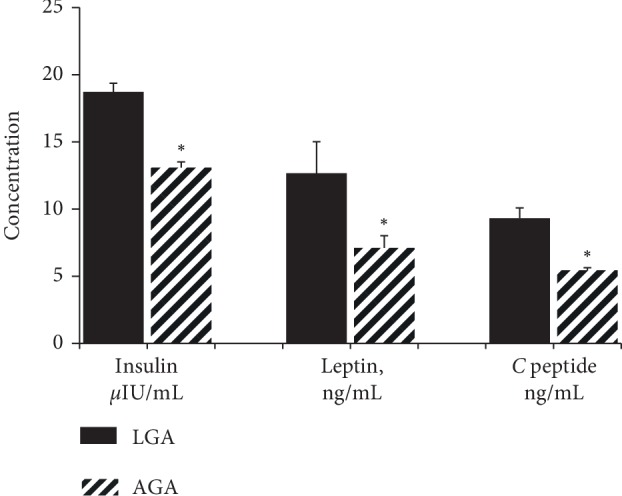
Comparison of cord blood leptin, insulin and C-peptide concentrations between newborns born LGA and AGA. Concentration of cord blood mean serum insulin, leptin and C-peptide in both large-for-gestational-age (LGA) and appropriate for gestational age (AGA) infants. ^∗^Denotes the statistical significance at *P* < 0.05.

Mean insulin concentration was significantly higher (*P* ≤ 0.001) in the LGA group (18.725 ± 0.644 *µ*IU/mL) compared to the AGA group (13.081 ± 0.428 *µ*IU/mL) (Figure 1). Same trend was observed in the leptin and C-peptide levels, where significantly higher concentrations of 12.670 ± 2.345 ng/mL and 9.318 ± 0.772 ng/mL (*P* < 0.001) was observed for the LGA groups compared to 7.108 ± 0.906 ng/mL and 5.439 ± 0.192 ng/mL in the AGA groups.

**Figure 2:**
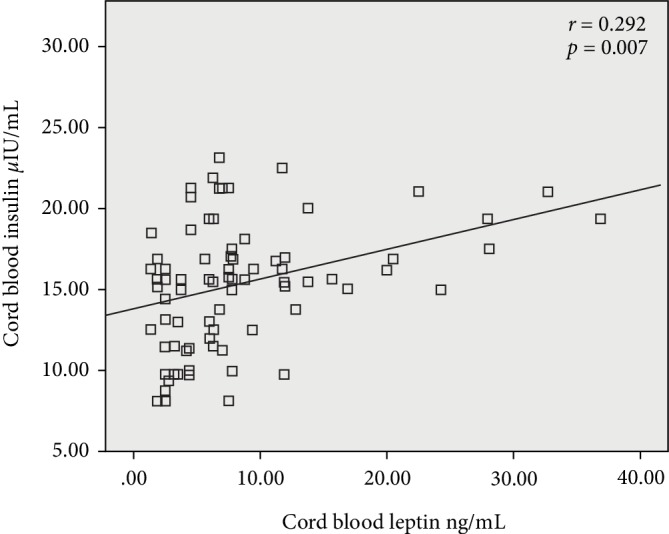
Correlation of leptin (ng/mL) with insulin levels (*µ*IU/mL) in the cord blood of all newborns.

When the whole study population was considered, leptin level was positively correlated with birth weight of the newborn (*r* = 0.296, *p* = 0.006) and maternal characteristics such as BMI before pregnancy (*r* = 0.225, *p* = 0.040), weight before pregnancy (*r* = 0.274, *p* = 0.012), C-peptide level (*r* = 0.344, *p* = 0.001), and insulin level (*r* = 0.292, *p* = 0.007). On the other hand, C-peptide level was positively correlated with birth weight of the newborn (*r* = 0.228, *p* = 0.037), insulin concentration in cord blood (*r* = 0.333, *p* = 0.002) and leptin concentration (*r* = 0.344, *p* = 0.001). Insulin concentration was correlated with only the leptin concentration (*r* = 0.292, *p* = 0.007) and the C-peptide concentration (*r* = 0.333, *p* = 0.002). Although the correlation between insulin and leptin was significant and positive as shown in Figure 2, it was a weak correlation. Hence, our results are slightly deviated from results reported in other studies where they report significant but strong correlations between insulin and leptin levels in cord blood suggesting the role of insulin in the stimulation of leptin production in the adipocyte.

## 4. Discussion

Relationships among neonatal anthropometry and its associations with fetal leptin, insulin and C-peptide concentrations and maternal characteristics in a Sri Lankan cohort of newborns who is at risk of childhood obesity was comprehensively examined in this study.

There is a limited amount of literature investigating the link between fetal metabolic markers of obesity and other anthropometric measures of neonatal adiposity, which could provide valuable insight into the fetal origins of future obesity and metabolic disease. Comprehensive reports from Sri Lanka on this topic are very limited. Populations differ with respect to both percentage fat mass and fat distribution, and in the relationship between body composition and morbidity. Since Asians have lower skeletal muscle mass, low bone mineral content and excess body fat for a given BMI compared to Caucasians [14], this study warranted an attempt to investigate the association between fetal metabolic factors on the birth of large-for-gestational-age infants with a special focus on fetal leptin, insulin and C-peptide concentrations and to compare those values with that of already published data to determine whether there are any deviations in the large-for-gestational-age infants born in Sri Lanka, compared to Caucasians, of whom the BMI cut-off values were derived.

According to published data, cord blood concentrations of leptin are associated with high BMI and increased central adiposity in children [15]. Catalano et al. reported that [16] compared to the offspring of lean mothers, offspring of obese mothers have an increased fat mass and percentage body fat. Thus, increased fetal adiposity and fetal insulin resistance are closely associated. Catalano et al. [17] also reported that the presence of obesity in the mother increases the incidence of macrosomic babies with an odd ratio of 1.68–1.73. In women without gestational diabetes mellitus, pre-pregnancy BMI and weight gain during pregnancy have been identified as the most influential characteristics affecting the birth weight of the offspring [18]. According to Mitra et al., maternal BMI and gestational age had significant positive correlations with birth weight (*p* < 0.05) [19]. These studies also suggested a direct influence of BMI and fat mass on increased maternal insulin secretion. These reports suggested that the high maternal insulin level affect the cord insulin and C-peptide levels and subsequently the birth weight of the infant [20].

As expected, leptin, a thoroughly investigated adipocytokine, was found in significantly higher levels in LGA newborns in this study when compared with AGA group (12.670 ± 2.345 ng/mL vs. 7.108 ± 0.906 ng/mL, *P* < 0.001), and correlated positively with birth weight. This finding is consistent with the results of previous studies [21]. Insulin levels were also higher in the LGA newborns (18.725 ± 0.644 *µ*IU/mL in LGA vs. 13.081 ± 0.428 *µ*IU/mL in AGA, *P* < 0.001) as shown earlier by others.Consistent with previous studies [22], this study also revealed that cord plasma leptin levels correlated significantly and positively with weight and adiposity at birth, and with cord plasma insulin levels. Thus we didn't see any significant difference between the data observed in our study and the data reported from the Caucasians.

Leptin is released into the circulatory system by the adipose tissue in proportion to the amount of lipid stores available in the body. It acts at the hypothalamic receptors and decreases food intake and increases energy expenditure [23]. According to the previous reports, during pregnancy and also in some forms of obesity, inhibited transport of leptin across the blood-brain barrier may lead to leptin resistance. It may also result from sequestration of bioactive leptin in the circulation by a soluble receptor [24, 25]. The positive associations in our study of cord blood leptin concentrations with birth weight and ponderal index might confirm the finding in previous investigations that leptin in fetal circulation may have been produced by the adipose tissue of the fetus [26].

Insulin plays a significant role in increasing the uptake of circulating glucose by fetal muscle and adipose tissue. Therefore, insulin is an important regulator of fetal growth. Prolonged hyperinsulinemia thus results in stimulation of fat deposition in the fetus and thereby fetal overweight [27]. Hyperinsulinemia was previously reported to have a long-term stimulatory effect on leptin production [28]. Therefore, it was hypothesized that fetal hyperinsulinemia in LGAs was considered as the primary cause for almost doubling the leptin levels by two factors, which mutually potentiate each other: increased fat deposition resulting in a greater potential for leptin synthesis, and stimulation of adipocyte *ob *gene transcription by insulin.

A significant correlation (*P* = 0.007) was observed between leptin and insulin levels in cord blood in the present study. Collectively, the present study provides evidence for a direct association between fetal leptin levels and birth weight in LGAs and AGAs. The correlation of cord blood insulin with birth weight is the result of the correlation between insulin and leptin. The data may suggest that, in addition to the direct anabolic action, part of insulin's effect on birth weight is mediated by leptin. It can also be speculated that insulin stimulates fetal adipocyte leptin synthesis. According to the results reported by Simental-Mendía et al. [29], mean insulin levels were 9.6 ± 4.9 *μ*U/mL in the cord blood of LGA neonates who had an average birth-weight of 4175 ± 197 g, and it was 52% higher than that was reported in the AGA group. In our study, we observed a median birth weight of 3700.00 kg (3550.00–3850.00) in the LGA group, and a higher insulin concentration (18.725 ± 0.644 *µ*IU/mL) which was 43% higher than the AGA group. These results are also consistent with what was reported earlier with Caucasians and we didn't observe any deviations in the Sri Lankan population.

Furthermore, Catalano et al. [30] also reported a positive correlation between fetal insulin resistance and fetal adiposity which support our finding that suggest that elevated body weight at birth might be the main risk factor involved in the development of hyperinsulinemia in the newborns. Our results based on measurement of cord insulin levels immediately after delivery, are in agreement with these findings and support the statement of a strong relationship between increase of insulin by fetus and the increase of body weight at birth.

Current study also shows that LGA newborns had significantly higher cord serum C-peptide levels in addition to higher leptin and insulin levels than AGA newborns, which is consistent with previous studies revealing that C-peptide levels in macrosomia were significantly higher than in AGA infants. C peptide is secreted by pancreatic beta cells in amounts equimolar with insulin, and its levels provide a direct indication of endogenous fetal levels of insulin despite the presence of maternal insulin antibodies.

However, longitudinal studies are necessary to clarify whether adipokines produced within the intrauterine and neonatal environment play an important role in fetal or neonatal programming of diseases occurring in adulthood, such as diabetes mellitus, hypertension, and coronary heart disease.

Strengths of this study include its study design which raises interesting questions regarding the interplay of leptin, insulin and C-peptide during the neonatal period and how it would influence the programming of obesity during infancy, childhood and may be adulthood. According to the recent notions that obesity may be influenced by maternal-fetal interactions, a prospective study following children from before birth would be of great use for the understanding of all parameters concerned. However, the main limitation of this study is the small sample size and we intend to continue the study to collect more data and investigate the relationships between adipocytokines and the future markers of metabolic syndrome.

## 5. Conclusion

According to the results of this study, significantly higher leptin, insulin and C-peptide concentrations were observed in infants born large-for-gestational-age compared to infants born appropriate for gestational age and they were also positively correlated to birth weight of the newborns and some of the maternal characteristics. These findings were comparable with results reported from other parts of the world. Hence, there may be other factors that contribute to the higher fat mass found in Asian populations and the previous finding that Asians have a higher body fat mass for BMI compared to other ethnic groups, is worth investigating in detail further. Finding this relationship as early as neonatal period will be useful in the prediction of risk factors and identification of infants who will be at risk of developing obesity related complications during childhood and adulthood.

## Figures and Tables

**Table 1:** Characteristics of maternal and neonatal demographics in appropriate and large for gestational age infants.

Variable	Total (*n* = 90)	AGA (*n* = 47)	LGA (*n* = 43)
*Characteristics of the child*			
Male/female	50/40	22/25	28/15
Gestational age at delivery (weeks)	39.00 (38.00–40.00)	39.00 (38.00–40.00)	39.00 (38.00–40.00)
Birth weight (kg)	3150.00 (2600.00–3550.00)	3150.00 (2800.00–3300.00)	3700.00 (3550.00–3850.00)^∗^
Birth length (cm)	51.00 (49.00–52.00)	51.00 (50.00–52.00)	52.00 (49.50–55.25)^∗^
Ponderal index	2.31 (2.15–2.52)	2.28 (2.21–2.41)	2.61 (2.20–3.01)^∗^

Circumferences (cm)			
Occipital frontal	34.00 (33.00–35.00)	34.00 (33.00–34.00)	35.00 (34.00–35.25)^∗^
Chest	33.00 (32.00–34.00)	33.00 (32.00–34.00)	34.00 (33.00–36.00)^∗^
Hip	33.00 (30.00–35.00)	34.00 (33.00–35.00)	30.00 (28.87–33.25)^∗^
Calf	11.50 (10.50–12.00)	12.00 (11.00–12.00)	12.00 (10.50–12.12)
Mid upper arm	11.00 (10.00–11.50)	11.00 (10.00–11.50)	12.00 (10.37–12.00)^∗^
Abdominal	33.00 (32.00–33.75)	33.00 (32.00–34.00)	33.00 (31.00–34.00)
Thigh	13.00 (12.00–14.00)	13.00 (12.30–13.00)	15.00 (14.00–16.00)^∗^

*Characteristics of the mother*			
Age	27.5 (23.25–31.75)	29.0 (25.0–32.0)	30.0 (24.5–33.0)
Weight before pregnancy (kg)	52.00 (48.00–57.63)	52.00 (49.00–56.00)	55.00 (49.42–69.00)^∗^
BMI before pregnancy (kg/m^2^)	21.61 (18.87–23.82)	21.67 (20.81–23.68)	23.14 (18.45–25.67)^∗^
Weight gain during pregnancy (kg)	11.25 (10.00–12.50)	10.50 (7.75–14.25)	12.00 (10.00–12.50)^∗^

AGA: Appropriate for gestational age, LGA: Large-for-gestational-age. Data are presented as median (interquartile range). Statistical analysis was performed using Kruskal–Wallis *T* test for the differences between AGA and LGA groups, ^∗^*P* < 0.05 between AGA and LGA.

**Table 2:** Characteristics of study subjects according to the sex.

	All neonates	Males (*n* = 50)	Females (*n* = 40)
Birth weight (g)	3101.90 ± 59.78	3229.11 ± 83.34	2955.13 ± 80.48^∗^
Length (cm)	50.60 ± 0.35	50.71 ± 0.56	50.49 ± 0.40
Ponderal index	2.366 ± 0.42	2.43 ± 0.06	2.29 ± 0.06

*Circumferences (cm)*			
Occipital frontal	33.63 ± 0.15	33.91 ± 0.20	33.27 ± 0.23
Chest	33.00 ± 0.19	33.46 ± 0.27	32.46 ± 0.28^∗^
Hip	32.14 ± 0.36	32.26 ± 0.51	31.92 ± 0.53
Calf	11.23 ± 0.14	11.38 ± 0.18	11.02 ± 0.20
Mid upper	10.75 ± 0.14	10.87 ± 0.20	10.58 ± 0.20
Abdominal	32.42 ± 0.21	32.72 ± 0.29	32.01 ± 0.30
Thigh	13.19 ± 0.15	13.33 ± 0.21	13.03 ± 0.20

Leptin concentration ng/mL	8.87 ± 0.84	8.28 ± 1.15	9.56 ± 1.21
Insulin concentration *µ*IU/mL	15.35 ± 0.43	15.50 ± 0.64	15.18 ± 0.57
C-peptide concentration ng/mL	6.87 ± 0.32	7.38 ± 0.50	6.27 ± 0.37

∗ denotes the statistical significance at *P* < 0.05

## Data Availability

The datasets used during the current study are available from the corresponding author on reasonable request.

## References

[1] Han J. C., Lawlor D. A., Kimm S. Y. S. (2010). Childhood obesity—2010: progress and challenges. *Lancet*.

[2] Dunger D. B., Ong K. K. (2005). Endocrine and metabolic consequences of intrauterine growth retardation. *Endocrinology and Metabolism Clinics of North America*.

[3] Hermann G. M., Dallas L. M., Haskell S. E., Roghair R. D. (2010). Neonatal macrosomia is an independent risk factor for adult metabolic syndrome. *Neonatology*.

[4] Happarty P. (2002). Placental regulation of fatty acids delivery and its effect on fetal growth—a review. *Placenta*.

[5] Patenaude J., Lacerte G., Lacroix M., Guillemette L., Allard C. (2017). Associations of maternal leptin with neonatal adiposity differ according to pregravid weight. *Neonatology*.

[6] Raj M. (2012). Obesity and cardiovascular risk in children and adolescents. *Indian Journal of Endocrinology and Metabolism*.

[7] Parker M., Rifas-Shiman S. L., Belfort M. B. (2011). Gestational glucose tolerance and cord blood leptin levels predict slower weight gain in early infancy. *The Journal of Pediatrics*.

[8] Wolf H. J., Ebenbichler C. F., Huter O. (2000). Fetal leptin and insulin levels only correlate in large-for-gestational-age infants. *European Journal of Endocrinology*.

[9] Tung W. K., Lin S. J., Hwang Y. S., Wu C. M., Wang Y. H., Tsai W.-H. (2009). Association of cord plasma leptin with birth size in term newborns. *Pediatrics & Neonatology*.

[10] Hauguel-de M. S., Lepercq J., Catalano P. (2006). The known and unknown of leptin in pregnancy. *American Journal of Obstetrics and Gynecology*.

[11] Tsai P., Yu C., Hsu S. (2004). Cord plasma concentrations of adiponectin and leptin in healthy term neonates: positive correlation with birth weight and neonatal adiposity. *Clinical Endocrinology*.

[12] Mitanchez D. (2010). Management of infants born to mothers with gestational diabetes. Paediatric environment. *Diabetes & Metabolism*.

[13] Hamidi A., Fakhrzadeh H., Moayyeri A., Heshmat R., Ebrahimpour P., Larjani B. (2006). Metabolic syndrome and leptin concentrations in obese children. *Indian Journal of Pediatrics*.

[14] Wang J., Thornton J. C., Russell M., Burastero S., Heymsfield S., Pierson R. N. (1994). Asians have lower body mass index (BMI) but higher percent body fat than do whites: comparisons of anthropometric measurements. *The American Journal of Clinical Nutrition*.

[15] Higgins M., Mc Auliffe A. (2010). A review of maternal and fetal growth factors in diabetic pregnancy. *Current Diabetes Reviews*.

[16] Catalano P. M., Presley L., Minium J., Hauguel-de Mouzon, S. (2009). Fetuses of obese mothers develop insulin resistance in utero. *Diabetes Care*.

[17] Catalano P. M., McIntyre H. D., Cruickshank J. K. (2012). The hyperglycemia and adverse pregnancy outcome study: associations of GDM and obesity with pregnancy outcomes. *Diabetes Care*.

[18] Retnakaran R., Ye C., Hanley A. J. (2012). Effect of maternal weight, adipokines, glucose tolerance and lipids on infant birth weight among women without gestational diabetes mellitus. *Canadian Medical Association Journal*.

[19] Mitra S., Misra S., Nayak P. K., Sahoo J. P. (2012). Effect of maternal anthropometry and metabolic parameters on fetal growth. *Indian Journal of Endocrinology and Metabolism*.

[20] Hou R. L., Zhou H. H., Chen X. Y., Wang X. M., Shao J., Zhao Z. Y. (2014). Effect of maternal lipid profile, C-peptide, insulin, and HBA1c levels during late pregnancy on large-for-gestational-age newborns. *World Journal of Pediatrics*.

[21] Tsai P. J., Yu C. H., Hsu S. P. (2004). Cord plasma concentrations of adiponectin and leptin in healthy term neonates: positive correlation with birthweight and neonatal adiposity. *Clinical Endocrinology*.

[22] Clapp J. F., Kiess W. (1998). Cord blood leptin reflects fetal fat mass. *Journal of the Society for Gynecologic Investigation*.

[23] Henson M. C., Castracane V. D. (2006). Leptin in pregnancy: an update. *Biology of Reproduction*.

[24] Highman T. J., Friedman J. E., Huston L. P., Wong W. W., Catalano P. M. (1998). Longitudinal changes in maternal serum leptin concentrations, body composition, and resting metabolic rate in pregnancy. *American Journal of Obstetrics and Gynecology*.

[25] Henson M. C., Castracane V. D. (2006). Leptin in pregnancy: an update. *Biology of Reproduction*.

[26] Krew M. A., Kehl R. J., Thomas A., Catalano P. (1994). Relation of amniotic fluid C-peptide levels to neonatal body composition. *American Journal of Obstetrics and Gynecology*.

[27] Fried S. K., Ricci M. R., Russell C. D., Laferrère B. (2000). Regulation of leptin production in humans. *The Journal of Nutrition*.

[28] Masuo K., Katsuya T., Ogihara T., Tuck M. L. (2005). Acute hyperinsulinemia reduces plasma leptin levels in insulin-sensitive Japanese men. *American Journal of Hypertension*.

[29] Simental-Mendia L. E., Castaneda-Chacon A., Rodriguez-Moran M., Guerrero-Romero F. (2012). Birth-weight, insulin levels, and HOMA-IR in newborns at term. *BMC Pediatrics*.

[30] Catalano P. M., Presley L., Minium J., Hauguel-de M. S. (2009). Fetuses of obese mothers develop insulin resistance in utero. *Diabetes Care*.

